# Case Report: Surgical resection of giant ventricular fibroma in an infant utilizing 3D imaging guidance

**DOI:** 10.3389/fcvm.2025.1492727

**Published:** 2025-04-10

**Authors:** Jin Lu, Yu Chen, Xingchen Lian, Peipei Chang, Ping Wen, Na Zou, Lin Ma, Yuhang Liu

**Affiliations:** ^1^ Department of Cardiovascular Surgery, Dalian Women and Children’s Medical Group, Dalian, China; ^2^ Graduate School, Dalian Medical University, Dalian, China; ^3^ Heart Center, The Sixth Affiliated Hospital of Haerbin Medical University, Haerbin, China

**Keywords:** cardiac tumor, fibroma, three-dimensional imaging, extracorporeal membrane oxygenation, infant

## Abstract

Primary cardiac tumors are extremely rare, with fibromas being one of the more prevalent type primary cardiac tumors in infants and children. Cardiac fibromas present a high risk of fatal arrhythmia and sudden death, hence more aggressive surgical treatment approaches are typically employed. However, certain populations, such as asymptomatic infants and young children in the early stages, require extra caution. We present the case of a patient with a giant fibroma of the heart detected during fetal development, who was followed up until the age of 5 months before undergoing surgical resection. Prior to surgery, we employed three-dimensional (3D) imaging technology to acquire a deeper understanding of the anatomical nature of cardiac tumors. We then devised a comprehensive surgical strategy to minimize the risk of damage to large blood vessels during surgery and maximize preservation of myocardial tissue. Following surgical resection of the tumor, cardiac dysfunction was managed with extracorporeal membrane oxygenation (ECMO) continuous adjuvant therapy, and conventional vasodilators such as dopamine and nitroglycerin were ad. The patient recovered well without any serious complications. This case highlights the significance of timely surgical intervention, combined with 3D imaging to develop a meticulous surgical plan and early use of ECMO to maintain cardiac function in patients with postoperative cardiac dysfunction. This can help to ensure the safety and effectiveness of giant cardiac fibromas resection in infants and young children.

## 1 Introduction

Primary cardiac tumors in children are extremely rare, with benign tumors accounting for about 90%, and among them, cardiac fibromas are the second most common after rhabdomyomas (1, 2). Despite being histologically benign, cardiac fibromas can pose significant clinical risks due to their location and interference with cardiac function, potentially causing fatal arrhythmias and sudden cardiac death; spontaneous resolution has not been observed. Early surgical resection is usually recommended (2–4). Though there is a consensus on the surgical indications for symptomatic patients with cardiac fibromas, the timing of surgery for asymptomatic infants and young children after birth remains contentious. For patients with cardiac fibromas requiring surgery, acquiring visual details of the anatomical structures before surgery is crucial for developing surgical strategies. Three-dimensional (3D) imaging is the computer-aided construction of a 3D digital model that simulate the visual experience of the human eye while seeing real-world objects. Compared to typical 2D images obtained from ultrasound, computed tomography (CT), magnetic resonance imaging (MRI), and other methods, 3D imaging conceptualizes complex anatomical structures and enables more accurate strategies and guides for surgical interventions (5). 3D imaging has been implemented in the development of preoperative surgical plans for cardiac tumors because of its substantial advantages in vascular visualization and its illustration of the relationships between adjacent tissues (6, 7). Extracorporeal membrane oxygenation (ECMO) is a life-support technology that temporarily replaces heart and lung function. ECMO is frequently applied in internal medicine for treatment of refractory heart and lung failure, particularly after middle- to high-risk cardiac surgeries. According to Deng et al. (8), early administration of ECMO before worsening into cardiac arrest may improve survival rates. We present here the case of an infant diagnosed with a giant fibroma of the heart during fetal development, who underwent successful cardiac tumor resection at 5 months of age. This case report demonstrates the successful implementation of 3D imaging in the development of a rigorous preoperative plan, the continuous use of ECMO to maintain cardiac function after surgery, thereby enabling patients to safely navigate the perioperative period and ultimately be discharged smoothly.

## 2 Case description

**Figure 1:**
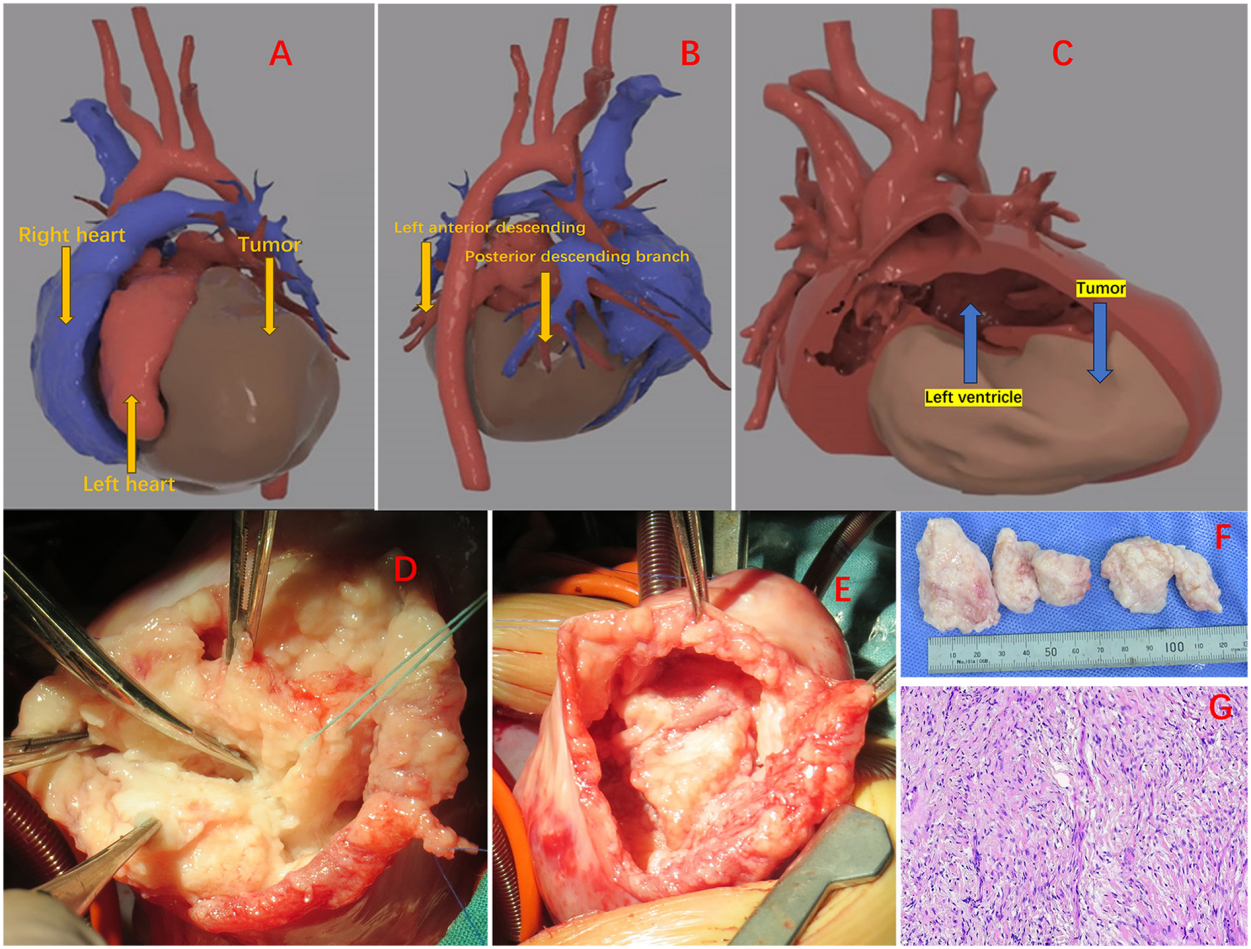
**(A)** The positional relationship of the tumor. **(B)** The mass is located between the anterior descending of the left coronary artery and posterior descending branches. **(C)** Massive mass pressing on the ventricular wall. **(D)** Start removing the tumor. **(E)** The cavity between the ventricular wall and the free wall after tumor resection. **(F)** Excised tumor. **(G)** Tumors are composed of fibrous tissue, with spindle shaped and short spindle shaped cells, elongated nuclei, and residual myocardial cells visible.

The patient's fetal ultrasound examination revealed a space-occupying lesion in the heart, and post-natal complete cardiac MRI revealed a huge cardiac fibroma, though there were no serious arrhythmias or cardiac dysfunction. There is no family history or genetic history, and the family considers surgical treatment temporarily unavailable. No special treatment was performed, and follow-up was only conducted outside the hospital. A follow-up cardiac ultrasound at 5 months of age revealed that the tumor was progressively becoming bigger, resulting in mild impaired left heart function. Consequently, surgical intervention was planned. To ensure that the surgical plan was precise, a CT scan was performed and 3D images were generated to dynamically display the location and volume of the mass (Figure 1A), and its relationship with various cardiac cavities and arteries. The tumor was found to be extending from the left posterior wall to the apex of the heart, and located between the anterior and posterior descending branches of the left coronary artery (Figure 1B). The tumor caused an increase in the volume of the heart tissue, resulting in compression and thinning of the left ventricular endocardium (Figure 1C). The surgery was performed by opening and suspending the pericardium and inserting a left heart drainage tube through the foramen ovale together with initiation of low-temperature extracorporeal circulation. Upon exposure, it was evident that the left ventricular wall is entirely occupied by a large tumor. The tumor was positioned between the anterior and posterior descending branches of the left coronary artery, preventing encroachment. A midline incision was made in the epicardium of the left ventricular wall to avoid penetration and preserve maximal myocardial adhesion (Figure 1D). Following tumor resection, a large cavity remained between the ventricular and free walls, requiring intermittent suturing to repair the heart (Figure 1E). Persistent ventricular tachycardia prevented the patient from detaching from extracorporeal circulation, prompting ECMO assistance and placement of a temporary pacing lead. Postoperatively, routine cardiac vasodilators such as nitroglycerin, dopamine, adrenaline, and antibiotics to prevent infection were administered. On the third day, bedside ultrasound showed an improvement in cardiac contractile activity, and arterial blood pressure showed an increase in pulse pressure, with good tolerance. Vital signs gradually stabilized. On the fourth day after surgery, ECMO evacuation and delayed thoracotomy were performed. The patient was weaned off the ventilator on the 16th day and be discharged on the 20th day. The pathological findings confirmed the presence of fibroma and infiltration into the myocardial wall (Figure 1F,G). At present, the patient's heart function is normal and the prognosis is good. The patient is undergoing regular follow-up examinations at the cardiac surgery clinic after surgery. So far, the recovery has been good and the tumor has not recurred.

## 3 Discussion

Cardiac fibroma is the second most common primary cardiac tumor in children. Despite benign histopathology, it is prone to fatal arrhythmia and sudden death. Based on previous reports and because of the involved risks, early surgical treatment is recommended (2, 3, 9). There are research reports that surgical treatment is advocated when severe clinical symptoms and hemodynamic damage occur (10, 11). It is difficult to grasp the timing of surgery for asymptomatic infants and young children. However, infants and young children have higher surgical risks due to immature organ development, fragile and tender tissues, small cardiac space, and difficult surgical procedures, worsened by their lower weight and age. Qian et al. (12) reported older children with relatively smaller fibroma sizes have a better prognosis after surgical resection. Yabrodi et al. (13) reported cases of survival after following ventricular tumor resection during neonatal period, although there was severe postoperative deterioration of left ventricular function with development of total heart block. In this case, they finally opted for cardiac resynchronization therapy and hospitalization lasted 6 months. There are reports that patients with severe ventricular outflow tract obstruction caused by tumor protrusion into the ventricle are treated with surgery, and there is a high risk of sudden death due to the involvement of conductive tissue after surgery (14). Determining the optimal surgical timing is critical and challenging for safely removing cardiac tumors in infants and young children. In our case, the patient's fetal ultrasound detected a cardiac mass lesion, prompting immediate transfer of the newborn to the CCU for the first assessment of cardiac function and tumor status. Subsequently, the patient entered the outpatient follow-up system. After a follow-up to up to 5 months of age, it was found that the tumor gradually increased in size, the ventricular function had slightly decreased with the occurrence of valve damage, but it had not yet caused serious consequences. Following an extensive examination, urgent surgical intervention was undertaken, and the patient recovered well following surgery. We suggest that patients be diagnosed early, enabling them to enter the hospital's follow-up management system as soon as possible, and attempting to age the patient before the tumor causes cardiac damage. The expeditious implementation of surgical treatment is recommended if there is a trend of deteriorating cardiac function during the follow-up period.

3D imaging is a computer-aided technology that creates stereoscopic images or movies that are more similar to the visual experience of the actual world. Compared to standard 2D pictures provided by CT and MRI, 3D imaging may convert medical imagi-ng information into visual narratives, transforming complex data and abstract concepts into easily accessible and tangible concepts (5). Currently, 3D imaging is able achieve large-scale personalized diagnosis and treatment, enhancing the visualization of anatomical relationships to provide guidance in the field of surgery (15). Recht et al. (16) utilized 3D imaging to examine chest vascular abnormalities, resulting in enhanced anatomical details and realistic quality. This not only facilitated the planning of surgical procedures for complex vascular abnormalities, but also facilitated communication among clinical doctors, trainees, and patients and their families. 3D imaging not only accurately depicts the complex interaction between blood vessels and contiguous tissues, but it also enables an enhanced understanding of the edges of cardiac tumors and their relationship with surrounding structures (6, 7, 17). The primary and most challenging aspect of surgery is complete removal of cardiac tumors without causing significant tissue damage while conserving as much myocardial tissue as possible, particularly in infants and young children. As a result, determining a more natural anatomical structure of the tumor and devising a complete preoperative strategy are critical guiding principles. In this case, we used 3D imaging in the form of a stereoscopic screen to fully display the tumor's size, location, and relationship with various cardiac chambers and large blood vessels from all angles, allowing us to have a more intuitive understanding of the anatomical structure in the surgical plan prior to surgery. In terms of specifics, it indicated the fragility of the left ventricular wall following tumor removal, offering guidance for a careful surgical procedure to avoid breaching the ventricular wall when the tumor is free and close to the left ventricular wall during surgery. At the same time, it was found that the tumor was located between the anterior and posterior descending branches of the left coronary artery. When the tumor was dissociated during surgery, the left coronary artery was meticulously avoided, which helped to restore ventricular function, ensure surgical safety, and reduce postoperative complications.

The excision of a large cardiac tumor during infancy and early childhood is a significant determinant in patient mortality because of severe myocardial stress and minimal preservation of postoperative myocardial tissue, culminating in left heart dysfunction. Maintaining left ventricular function and ensuring safe passage through the perioperative period is the key to successful surgical treatment. Traditionally postoperative patients are initially administered high-dose vasoactive drugs to maintain cardiac function, and ECMO should be used as an adjunct supportive procedure when cardiopulmonary failure cannot be resolved (18, 19). There has been significant development in pediatric ECMO, with advances in circuit maintenance, nutritional support, and clinical decision-making, reducing much of the complications that were once considered insurmountable (20). It has now become a valuable tool for treating critically ill children with cardiac and pulmonary failure, especially those who cannot escape extracorporeal circulation after congenital heart surgery (20). ECMO is becoming increasingly prevalent and safe in pediatric usage. Therefore, we anticipated before surgery that the patient might be unable to escape extracorporeal circulation due to left ventricular failure, and devised a strategy for postoperative immediate integration with ECMO aided treatment. As expected, this continuous treatment helped in avoiding the instability of vital signs caused by postoperative heart function decline, allowing patients to smoothly navigate the perioperative period of heart failure.

## 4 Conclusion

In summary, grasping the appropriate surgical timing, combining 3D imaging with a meticulous surgical plan, and using ECMO as early as possible to maintain cardiac function in patients with postoperative cardiac dysfunction can further ensure the safety and effectiveness of the removal of giant cardiac fibromas in infants and young children.

## Data Availability

The original contributions presented in the study are included in the article/Supplementary Material, further inquiries can be directed to the corresponding authors.
